# Ultrasound‐assisted solubilization of calcium from micrometer‐scale ground fish bone particles

**DOI:** 10.1002/fsn3.2696

**Published:** 2022-01-29

**Authors:** Juanjuan Guo, Siliang Zhu, Hongbin Chen, Zongping Zheng, Jie Pang

**Affiliations:** ^1^ College of Oceanology and Food Sciences Quanzhou Normal University Quanzhou China; ^2^ College of Food Science Fujian Agriculture and Forestry University Fuzhou China; ^3^ Key Laboratory of Inshore Resources Biotechnology (Quanzhou Normal University) Fujian Province University Quanzhou China

**Keywords:** calcium release, fish bone particles, mineralized collagen fibrils, ultrasonication

## Abstract

In order to promote the extraction of biological calcium from fish bone, ultrasonication was used to process micrometer‐scale fish bone particles (MFPs) and investigate the mechanism of action in relation to bone structure. With ultrasonication treatment (300 W, 60°C, 2 h), the content of calcium release increased by 25.6%. Calcium release reached 94.0% of total calcium after 24‐h treatment. The surface of the MFPs was significantly damaged by ultrasound‐induced cavitation, resulting in holes and separation of the layered structure. X‐ray diffraction (XRD) and Fourier transform infrared (FT‐IR) analysis demonstrated that the crystalline structure of hydroxyapatite was disrupted, the triple helical structure of mineralized collagen fibrils (MCFs) was loosened, and hydrogen bonding in collagen decreased, facilitating the release of hydroxyapatite crystals. Thus, ultrasonication may be a practical alternative to nanomilling for industrial processing of waste fish bones to produce soluble calcium as an ingredient in calcium supplements and supplemented foods.

## Graphical abstract

Our work was to promote calcium release from micrometer‐scale fish bone particles (MFPs) by ultrasonication and investigate the mechanism of action in relation to bone structure. Ultrasonication at 300 W, 60°C and for 2 h increased calcium release by 25.6%, and calcium release reached 94% of total calcium after 24‐h treatment. Furthermore, the crystalline structure of hydroxyapatite was disrupted, the triple helical structure of mineralized collagen fibrils (MCFs) was loosened, and hydrogen bonding in collagen decreased, facilitating the release of hydroxyapatite crystals.

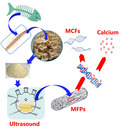

## 1 INTRODUCTION

Leftovers (e.g., fish bone, skin, and fish viscera) from fish processing are causing increasing environmental problems with the rapid growth of the global fishing industry (Marliana et al., 2015). These leftovers, however, are rich in nutrients, such as proteins, lipids, and minerals. Fish bones are a major by‐product of fish processing and are a potential biological source of natural inorganic calcium (Xu et al., 2020). Some research has been undertaken on the utilization of calcium from fish bones, for example, using it to make calcium‐fortified food. However, conversion of fish bones into a bioavailable form of calcium that is easily absorbed from the digestive system is difficult, and there is a clear need for effective processing methods to achieve this industrially (Li et al., 2020; Yin et al., 2015).

Mackerel (*Trachurus trachurus*), a low‐value bulk fish, has been used as a source of fish bones for processing to extract calcium (Ferraro et al., 2013). Calcium in Mackerel bones is in the form of hydroxyapatite (HA) and β‐tricalcium phosphate (Terzioglu et al., 2018). The main components of fish bones are the inorganic phase (calcium phosphate, CaP) ceramics (mainly hydroxyapatite (HA) crystals) and the organic/protein phase (mineralized collagen fibrils, MCFs), which form a complex matrix (Xu et al., 2020). The MCFs (mainly collagen‐Ⅰ, 90%) have a triple helical collagen structure and act as an adhesive, strongly binding to hydroxyapatite, and giving the bone stiffness and resilience. Since hydroxyapatite crystals are embedded in the collagen matrix, it is extremely difficult to extract calcium from them. The common current method to promote calcium release from fish bones is grinding the material into nanometer‐scale particles. For example, high‐energy wet ball milling can grind fish bones into particles with an average size of 110 nm, which increased the degree of calcium dissolution from 0.7% to 27.4% (Yin et al., 2016). Nanogrinding degrades the collagen‐fiber network structure and breaks down the hydroxyapatite crystals by collision and crushing (Eskin et al., 2005). Thermal treatment is another method to promote calcium release from fish bone through degradation of the collagen fiber matrix and reducing the mechanical strength of the bone. Heating effectively promoted calcium release from previously ground nanoscale fish bone particles (Jiang et al., 2020; Zhang et al., 2016). Therefore, degradation of the collagen matrix is an effective method to release calcium from fish bones. However, nanomilling is costly, and a feasible alternative technology to increase calcium release from microscale fish bone particles is indispensable.

Ultrasound waves in a liquid system generate cavitation bubbles, which grow larger by absorbing gas or vapor, then collapse, releasing the absorbed energy as heat and breaking up any solid material present, which has been reported that can disrupt the triple helical structure of collagen and facilitate the decalcification of bones in the medical field (Amiri et al., 2018). Ultrasonication has been used to extract gelatin from animal skins, by destabilizing the collagen structure and loosening the triple helical structure (Ali et al., 2018b). Ultrasound treatment has been used to extract collagen‐Ⅱ from chicken sternal cartilage; the triple helical structure was partially denatured after longer treatment times (Akram & Zhang, 2020). Ultrasound treatment has also been used for decalcification of bones, or organs in the medical field, indicating that it should be able to release calcium from fish bones (Chen et al., 2007; Milan & Trachtenberg, 1981). However, to our knowledge, there has been no report on the extraction of soluble calcium from fish bones using ultrasonication.

In this study, fish bone particles in the micrometer size range were made from Mackerel bones by ultrafine friction milling. Changes in the physicochemical properties (particularly calcium release and micromorphology) of fish bone particles during ultrasound treatment were investigated and compared. The mechanism by which ultrasound treatment promotes calcium release from fish bone particles was analyzed to help provide a theoretical basis for the practical industrial extraction of soluble biological calcium from fish bones.

## 2 MATERIALS AND METHODS

### 2.1 Materials

Mackerel (36 cm length, 457 g/fish) were provided by Ruifang Food Co., Ltd., Quanzhou, China. Mackerel were filleted and deboned using a roll‐type meat separator (YBYM‐6004‐B, Yingbo Food Machinery Co., Ltd.), and fish backbones were collected. The fish backbones were cleaned two to three times using deionized water to remove blood and flesh, and then stored at −20°C. All reagents used were of analytical grade from local suppliers.

### 2.2 Preparation of coarse fishbone particles (CFPs) and micrometer fishbone particles (MFPs)

Frozen fish backbones were thawed at room temperature, cut with a knife into pieces 5–10 cm long, then immersed in 40% aqueous Na_2_CO_3_ at a ratio of 1:3 (w/v), and autoclaved at 121°C (100 kPa) for 1 h to remove connective tissue and fat. The cooled, separated vertebrae were cleaned two to three times using deionized water and dried at 105°C for 1 h.

Coarse fishbone particles (CFPs): The fish vertebrae were ground using a high‐speed pulverizer (FW100, 50 Hz, Tester Instruments Co. Ltd) at a speed of 4,500 rpm for 60 s.

Micrometer fishbone particles (MFPs): The CFPs were further ground using an ultrafine friction grinder (PX‐MFC 90D, WIGGENS) at a speed of 3,000 rpm for 45 s.

### 2.3 Determination of particle size

The mean particle size and particle size distribution of CFPs and MFPs were determined by laser light‐scattering, using a Microtrac S3500 analyzer (Microtrac Inc.). The particle sizes of CFPs and MFPs in the micrometer range (1–1,000 μm) were measured, and the results were analyzed with Microtrac S3500 software using a Mie scattering model. Raw data were drawn with Origin 8.5 software.

### 2.4 Determination of content of calcium release

The content of calcium release was determined under an in vitro*‐*simulated digestion system to digest samples according to a previously described method (Zhang et al., 2017). Samples (3 g) were dissolved in simulated gastric fluid (SGF, 100 ml). SGF was composed of KCl (6.9 mM), NaCl (42.7 mM), CaCl_2_.2H_2_O (0.15 mM), NaHCO_3_ (25 mM), KH_2_PO_3_ (0.9 mM), MgCl_2_(H_2_O)_6_ (0.12 mM), (NH_4_)_2_CO_3_ (0.5 mM), and HCl (15.6 mM) (Brodkorb et al., 2019). The pH was adjusted to 3.0 ± 0.2 using 6 mol/L HCl and porcine pepsin solution (0.5 ml, 3200 U/mg, Sigma‐Aldrich) was added to achieve an activity of 2,000 U/mL in the final digestion mixture. The mixture was incubated in a constant temperature shaker at 37°C and 140 rpm/min for 2 h, and then, the samples were centrifuged at 3996 *g* for 20 min and the supernatant was filtered and diluted with deionized water. Additionally, the factors in simulated gastric fluid including enzyme (porcine pepsin, other enzymes), pH (pH = 1, 3, 5 and 7) and digested time (0, 1, 2, 4, 6, and 8 h), were further investigated to explore the optimal factors. The kinetics of ultrasonic treatment time was also conducted. The calcium concentration was measured using an atomic absorption spectroscope (A3, Beijing Purkinje General Instrument Co. Ltd) as the method of GB/T 5009.92‐2003; the determination parameters were set to wavelength of 422.7/nm, height of burning head 6/mm, lamp current of 3/mA, flame Air‐Acetylene, acetylene flow rate of 1500 L/min.

### 2.5 Determination of chemical components

The contents of moisture, ash, fat, protein, phosphorus, and heavy‐metal ions including, Pb, As, Hg, Cd, and Cr, were detected according to Jin Zhang and Pham Viet Nam's methods (Nam et al., 2019; Zhang et al., 2016, 2017). The inductively coupled plasma‐optical emission spectrometry (ICP‐OES) analysis was performed using a PerkinElmer Optima 4300 DV spectrometer.

### 2.6 Ultrasound‐assisted calcium extraction

Control group: MFPs (3 g) were added to 100 ml simulated gastric fluid as described above without adding a digestive enzyme, and the mixture was incubated as described in Section 4. Ultrasound‐treated fish bone particles (U‐MFPs): MFPs (3 g) were added to 100 ml simulated gastric fluid as described above without adding a digestive enzyme, and the mixture was prepared using an ultrasonic extractor SCIENTZ‐ⅡDM (Ningbo Scientz Biotechnology Co., LTD) equipped with an amplitude transformer‐Φ6. To optimize the ultrasound treatment conditions, ultrasound treatment was performed at a fixed frequency (20 kHz), but the power (100, 300, and 500 W) at temperature 37°C for 2 h, temperature (37, 60, and 85°C) at power 300 W for 2 h and time (0, 1, 2, 3, 4, 6, 8, 10, 12, 14 and 24 h) at 300 W, 60°C were varied. The processing mode was tip‐type, and the extractor was operated in a pulsed mode, with 5‐s sonication and 5‐s resting time, in order to avoid over‐heating of the reaction system.

### 2.7 Mathematical fitting of calcium release with ultrasonic treatment

The Higuchi equation (Equation 1) and zero‐order kinetic function (Equation 2) were used to curve‐fit calcium release after ultrasonic treatment. The mathematic models were as follows:

(1) $$R\left(t\right)=R_{0}+kt^{1/2}$$

(2) $$R\left(t\right)=R_{0}+kt$$

where *R*_0_ is the initial calcium release of MFPs (mg/g_MFPs_), *k* is a calcium release constant, which can be determined by linear regression analysis between *R*(*t*) and ultrasonication time^1/2^, *t* is the ultrasonication time (h), and *R*(*t*) is the calcium release after a given ultrasonication time (mg/g_U‐MFPs_). A higher *k* value corresponds to a higher overall release rate.

### 2.8 Characterization of CFPs, MFPs, and U‐MFPs

#### 2.8.1 Morphological observation

The microstructures of CFPs, MFPs and ultrasound treated samples (U‐MFPs) were visualized using a scanning electron microscope (SEM; JSM‐6380LV, Tokyo, Japan). Before scanning, samples were mounted on a bronze stub and sputter‐coated with gold. The instrument settings were as follows: accelerating voltage, 5.0 kV, and magnification, ×50,000.

#### 2.8.2 Fourier‐transform infrared spectroscopy (FTIR)

FTIR was used to analyze the functional groups present in inorganic hydroxyapatite and organic mineralized collagen fibrils (MCFs). The samples were vacuum freeze‐dried and recorded using a Nicolet™ 360 FTIR spectrometer (Thermo Fisher Scientific, Waltham, MA). A spectral range from 500 to 4000 cm^‐1^ with a resolution of 2 cm^−1^ was analyzed.

#### 2.8.3 X‐ray diffraction (XRD)

The samples were vacuum freeze‐dried, and crystal structural analysis of hydroxyapatite and MCFs was performed using an X‐ray diffractometer (Rigaku TTR‐III, Rigaku) with a Cu*K*α X‐ray source (*λ* = 1.54178, generator voltage of 40 kV, incident current of 200 mA). Scanning was carried out from 5 to 50° at a scan rate of 0.02 deg/s.

### 2.9 Statistical analysis

All experiments were performed in triplicate with a completely randomized design. Analysis of variance (ANOVA) and regression was accomplished using Statistical Analysis System software (SAS Institute). Differences between mean values were determined by Duncan's multiple range tests.

## 3 RESULTS AND DISCUSSION

### 3.1 Particle size and morphology of CFPs and MFPs

**FIGURE 1:**
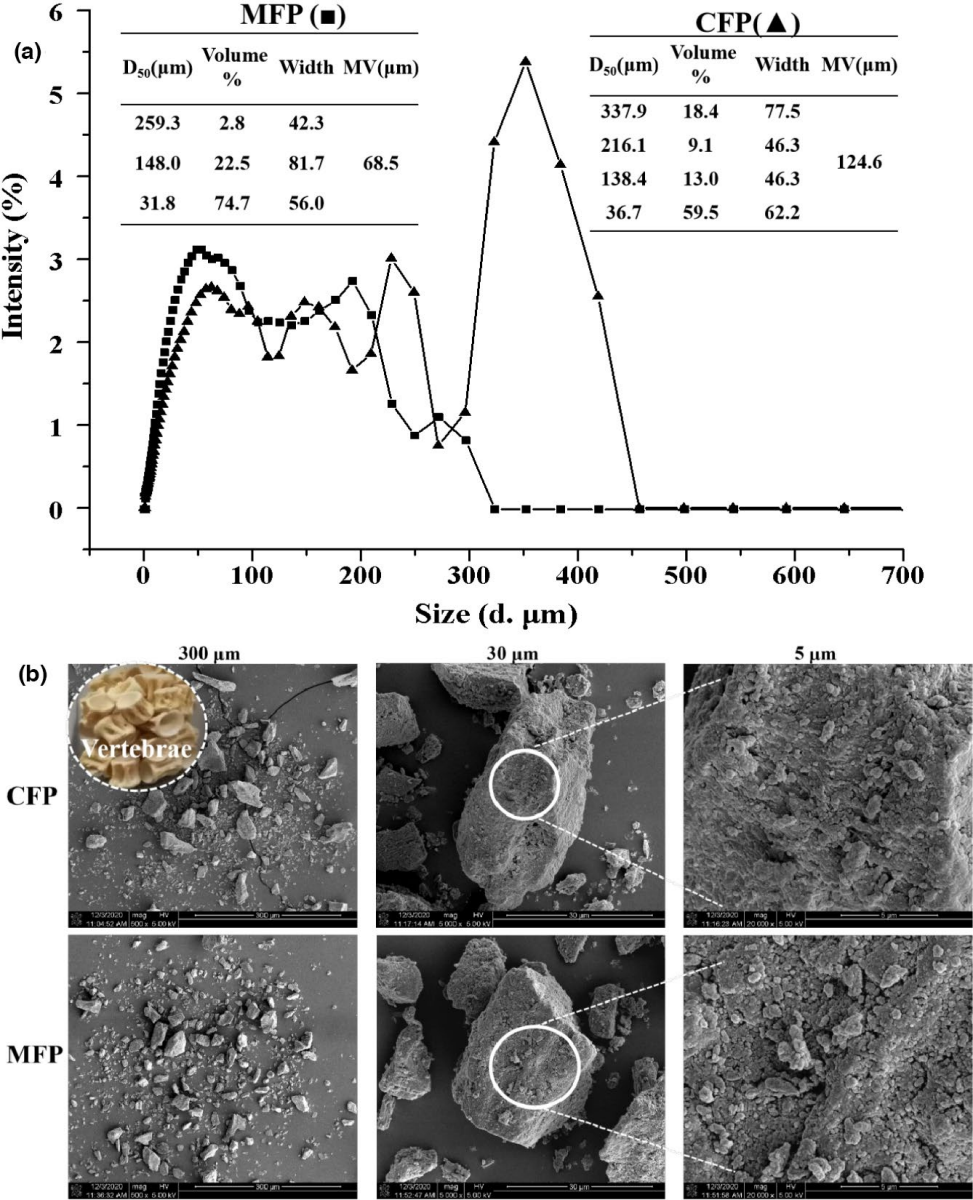
Particle size of CFPs and MFPs. (a) Particle size distribution and mean particle size (μm) of CFPs and MFPs. (b) Scanning electron microscopy images for CFPs and MFPs

The mean diameter of the volume distribution (MV) was 124.6 μm for CFPs, markedly higher than that of MFPs (MV = 68.5 μm) (Figure 1a). The distribution peak at D_50_ = 337.9 μm for CFPs was absent after ultrafine friction treatment and the size distribution of MFPs contained few particles larger than 300 μm. The volume % of particles larger than 200 μm reduced from 27.5% in CFP material to 2.8% in MFP. The ultrafine friction treatment effectively reduced the particle size of ground fish bones. The reduced particle size after ultrafine friction treatment was also apparent from SEM imaging (Figure 1b). However, the surface morphology of CFPs and MFPs did not appear significantly different, suggesting that ultrafine friction treatment did not disrupt the internal structure of the particles, which is necessary to efficiently release soluble calcium. Compositional analysis of CFPs and MFPs (Table 1) revealed some significant differences. The contents of fat, protein, and phosphorus decreased after ultrafine friction treatment. The contents of moisture (2.7%–3.0%) and calcium (152–175 mg/g) increased in MFPs. What is more, the contents of heavy metal ions were significantly decreased after ultrafine friction treatment, and within the range of Chinese standards. A previous report demonstrated that reduction in fish bone particle size facilitates calcium release, because of larger specific surface area and increased porosity, which facilitates the access of acid to the inorganic phase of the particles (Jeong et al., 2013). For example, nanomilling increases calcium release from fish bone particles compared with micromilling (Li et al., 2020; Yin et al., 2015). In addition, thermal treatment of nanoscale fish bone particles was more effective in promoting calcium release than that on microscale particles (Zhang et al., 2016). However, nanomilling is costly and a feasible alternative technology to increase calcium release from microscale fish bone particles is indispensable.

**TABLE 1:** Component analysis of fish bone, CFPs, and MFPs

	Moisture (%)	Ash content (%)	Fat content (g/100 g)	Protein (g/100 g)	Calcium (mg/g)	Phosphorus (mg/g)	Ca:P
Fish bone	2.406 ± 0.262^b^	79.631 ± 0.237^a^	5.561 ± 0.132^ab^	1.202 ± 0.080^ab^	137.808 ± 2.429^c^	116.907 ± 8.375^b^	1.179:1^b^
CFP	2.662 ± 0.011^b^	79.239 ± 0.356^a^	5.684 ± 0.338^a^	1.211 ± 0.081^a^	152.072 ± 2.655^b^	127.858 ± 5.940^a^	1.189:1^b^
MFP	3.039 ± 0.095^a^	78.528 ± 0.139^ab^	5.470 ± 0.007^b^	1.122 ± 0.001^b^	174.894 ± 0.460^a^	114.386 ± 8.308^b^	1.530:1^a^

	Pb (mg/kg)	As (mg/kg)	Hg (mg/kg)	Cd (mg/kg)	Cr (mg/kg)
Fish bone	0.701 ± 0.050^a^	0.79 ± 0.010^a^	–	0.017 ± 0.003^b^	0.1 ± 0.030^b^
CFP	0.239 ± 0.050^b^	0.61 ± 0.010^ab^	–	0.030 ± 0.003^a^	0.34 ± 0.030^a^
MFP	0.146 ± 0.050^c^	0.48 ± 0.010^b^	–	0.029 ± 0.003^a^	0.22 ± 0.030^ab^
Chinese standard	≤1.0	≤0.5	≤1.0	≤0.1	≤2.0

Note

Results are presented as mean ± *SD* (*n* = 3). Different lowercase letters within the same column indicate significant differences among fish bone, CFP, and MFP (*p* < .05).

Chinese standard: GB 2762‐2017

### 3.2 Calcium release

#### 3.2.1 Effect of digest factors

**FIGURE 2:**
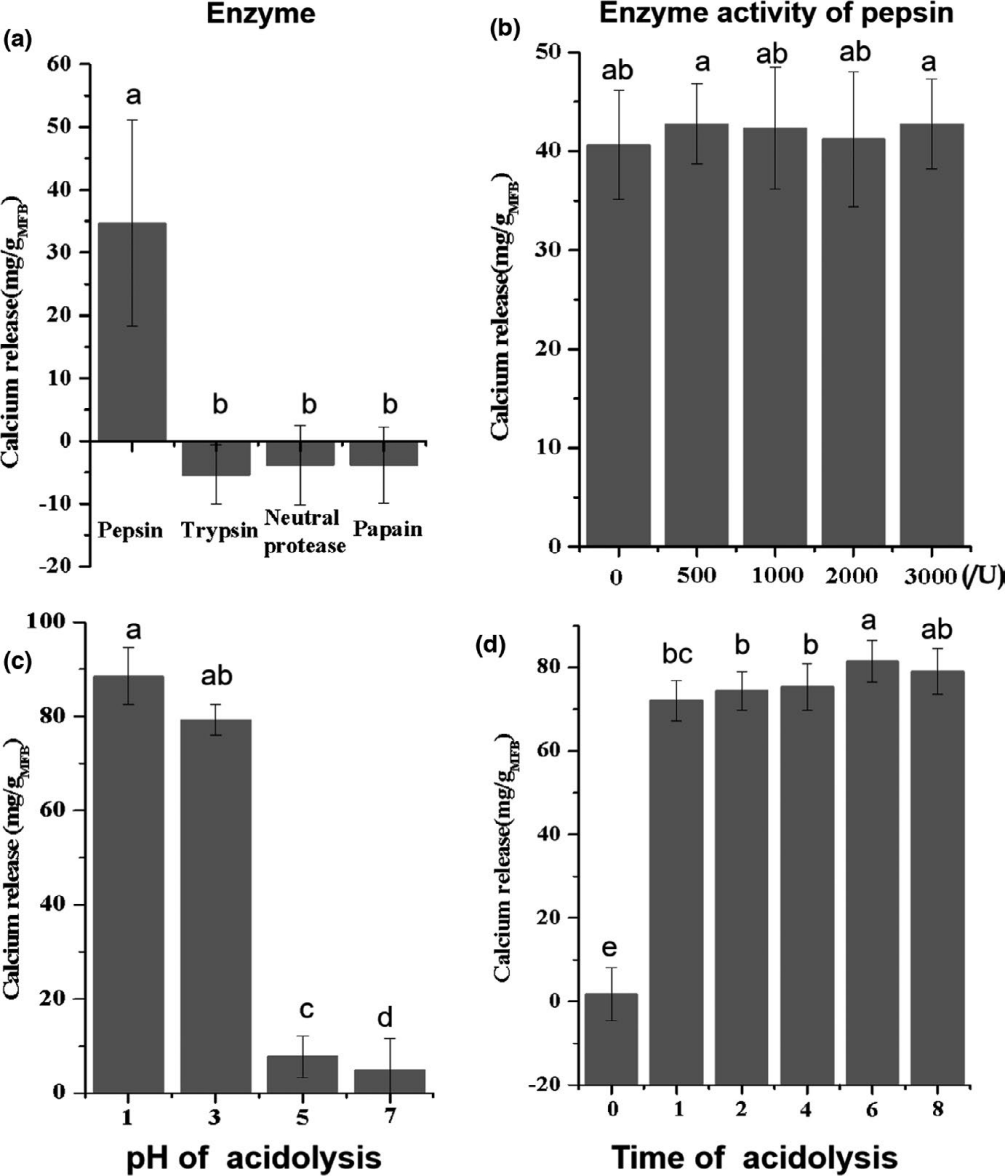
Effects of acid hydrolysis on the calcium release mean size of MFPs. a, b, c, and d represent enzyme, enzyme activity, pH, and acidolysis time, respectively

The assessment of calcium release was conducted in a simulated gastric system (Li et al., 2020; Yin et al., 2015); thus, the factors that might affect the calcium release in the simulated gastric system were explored. The pH (pH = 3) and digestive enzyme (pepsin) were the main factors in the gastric system that affected calcium release, so the effects of different enzymes and pH were investigated. Pepsin and other enzymes (trypsin, neutral protease, and papain) were applied to digest MFPs. However, enzymes other than pepsin, operating at their pH optima, had no effect on calcium release (Figure 2a). Moreover, the amount of calcium release was not significantly different with additions of pepsin between 0 and 3,000 U/mg, indicating that calcium release in the gastric system depends on acid hydrolysis, rather than on pepsin (Figure 2b). Varying the pH (Figure 2c) indicated that little calcium was released at pH above 3, but that pH 1 was only slightly more effective. The amount of calcium release did not change much after acid hydrolysis between 1 and 8 h (Figure 2d). Therefore, subsequent calcium release measurements were conducted in the simulated gastric system at pH 3, without pepsin present.

#### 3.2.2 Effect of ultrasound

**FIGURE 3:**
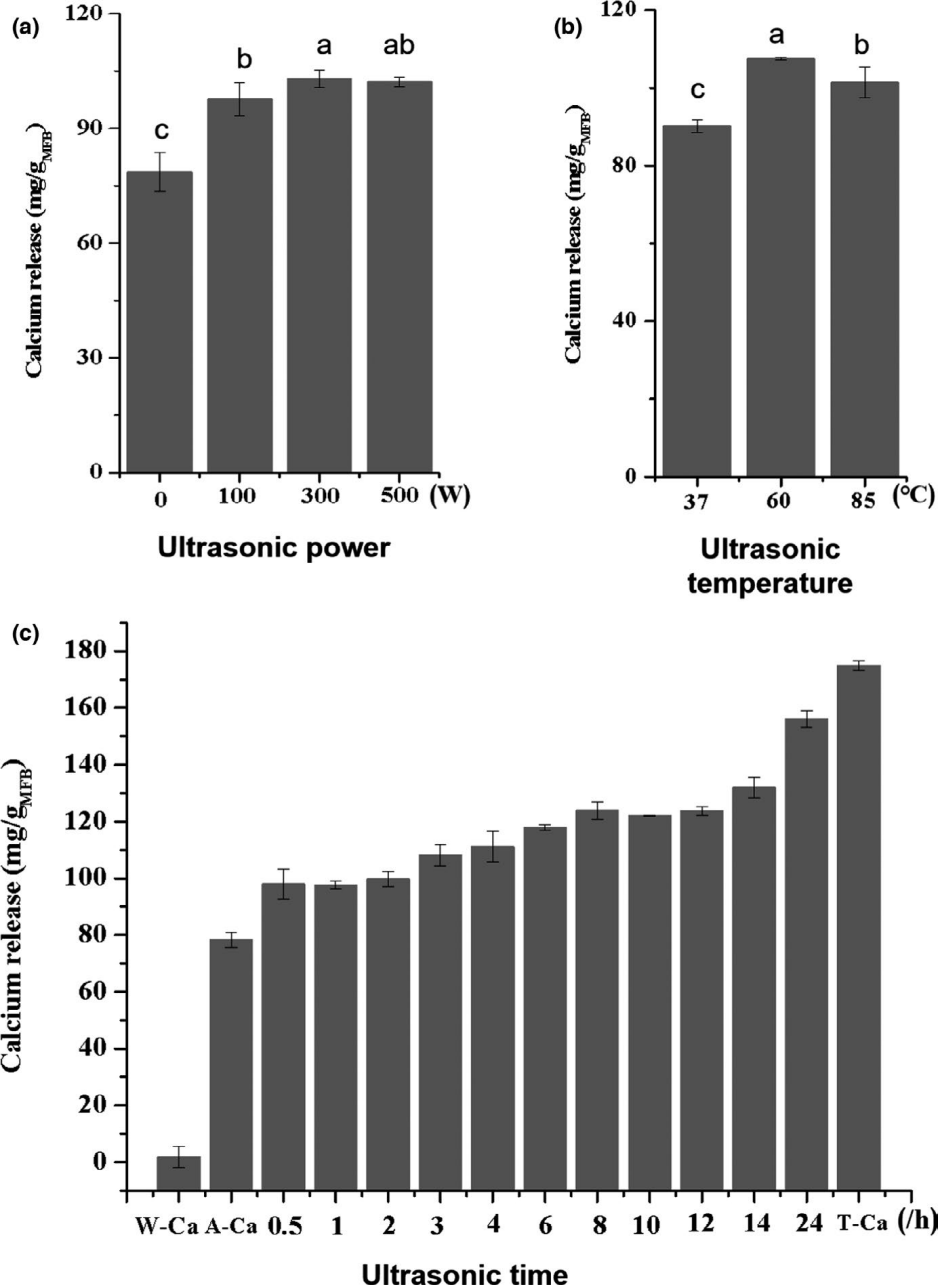
Effects of ultrasonic treatment on the calcium release mean size of MFPs. a, b, and c represent ultrasonic power, temperature, and time, respectively. W‐Ca: content of calcium release with water treatment; A‐Ca: content of calcium release with acid hydrolysis treatment; T‐Ca: total content of calcium

Since ultrasonication can disrupt the triple helical structure of collagen and facilitate the decalcification of bones, the parameters of ultrasonic power, temperature, and treatment time were systematically varied to optimize ultrasound‐assisted acid hydrolysis and promote calcium release from MFPs. The amount of calcium released increased with ultrasonic power, and at 300 W, was about 1.3 times that of the no‐ultrasonication control (*p* < .05, Figure 3a). Ultrasonic treatment temperature also influenced calcium release, reaching a maximum of 107.5 mg/g MFPs (64%) at 60°C (Figure 3b). However, this figure was only slightly higher than that achieved at the 300 W power setting, indicating that temperature has minimal influence on ultrasonication‐assisted calcium release. This is in contrast with a report that thermal treatments increase calcium release from fish bone particles during high‐energy wet ball milling (Zhang et al., 2016). This appears to be because the particles in this study were of micrometer scale, rather than nanometer scale (Jiang et al., 2020). Calcium release significantly, but very gradually, increased with increasing treatment time (Figure 3b), reaching 94.0% after 24 h at 300 W and 60°C, compared with 53.0% after 0.5 h.

#### 3.2.3 Kinetics of ultrasonic treatment

The relationship between calcium release and ultrasonication time was fitted to the Higuchi function (Section 2.2.5, Equation 1) and the zero‐order kinetic function (Equation 2) (Table 2). The Higuchi function had a better correlation (*R* ^2^ > 0.99), similar to that in a previous report on the effect of thermal treatments on calcium release from fish bones during high‐energy wet ball milling (Zhang et al., 2017). These data suggest that the effects of ultrasonic treatment on calcium release may be similar to that of high‐energy wet ball milling.

**TABLE 2:** Equation parameters fitted to calcium release data for MFPs during ultrasonic treatment

Equation	Measured *R*_0_	Fitted parameters		*R* ^2^
		*R*_0_	*k*	
Higuchi function (Equation 1)	78.6723	82.3966	13.7125	0.9941
Zero‐order kinetic function (Equation 2)		95.3910	2.6008	0.9010

### 3.3 Characteristic of ultra‐sonication of MPFs

#### 3.3.1 Surface morphology

**FIGURE 4:**
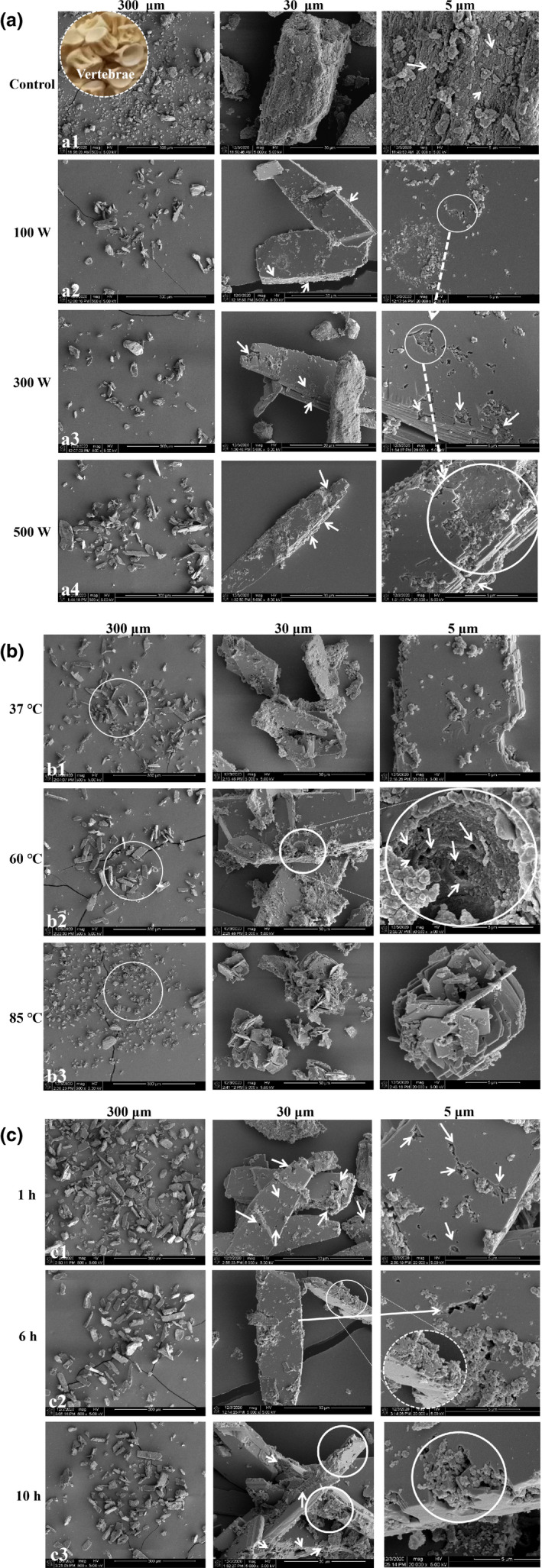
Effects of ultrasonic treatment on the surface morphology of MFPs. a, b, and c represent ultrasonic power, temperature, and time, respectively. Arrows show the structural changes in density and aperture

The surface morphology of MFPs after different ultrasonic power, temperature, and time treatments is shown in Figure 4. The fish bones used were vertebrae (inset in Figure 4a1). Without ultrasonication, the surface of the MFPs was dense and ordered, with stacked layers of planar mineralized collagen fibrils (MCFs) and much smaller hydroxyapatite crystals. The MCFs were well ordered along the longitudinal axis, and hydroxyapatite crystals filled the intervals between adjacent MCFs in a staggered arrangement (Figure 4a1). After different ultrasonic power treatments, multilevel hierarchical structures were clearly visible. The MCFs appeared to be arranged in parallel, forming flat sheets, which had been partially separated by the ultrasonic treatment. It also appeared that many of the hydroxyapatite crystals had been removed, making the layered structure more clearly visible (Figure 4a2, a3, a4 in 300 μm and 30 μm magnification). It appears that disruption of the layered structure facilitates the release of hydroxyapatite crystals. Moreover, the MCFs displayed holes and damage to their edges, which became more prominent with increased ultrasonic power (Figure 4a2, a3, a4 in 5 μm magnification). Ultrasound waves cause the creation, growth, and collapse of bubbles in liquid, known as cavitation (Liew et al., 2015). The diffusion of solvent vapor into the bubbles leads to their growth and implosive collapse, producing a localized hotspot by adiabatic compression (Bastami & Entezari, 2012). The hot spots momentarily reach very high temperatures (5000–25,000 K) and can break intramolecular chemical bonds, especially in organic compounds (Neppolian et al., 2008). Therefore, it appears that cavitation disrupts the surface of MFPs, especially the organic collagen, to facilitate the release of hydroxyapatite crystals.

After ultrasonic treatment at different temperatures, the changes in morphology were similar (Figure 4b) to those in Figure 4a, but more pronounced. With treatment at 60°C, the damage was more severe and the holes were bigger (Figure 4b2). However, after treatment at 85°C, the particles appeared much more extensively disrupted, with the layered structure mostly separated and the layers broken into much smaller pieces, although calcium release did not increase further (Figure 4b3, Figure 3b). Increasing treatment time (at 300 W and 60°C) resulted in similar changes to those in Figure 4a, which became gradually more pronounced (Figure 4c). Therefore, ultrasonic cavitation could disrupt the layered structure of the MFPs resulting in the appearance of holes in the layers and the near disappearance of hydroxyapatite crystals from the outside of the particles and from between the separated layers.

#### 3.3.2 FT‐IR spectra

**FIGURE 5:**
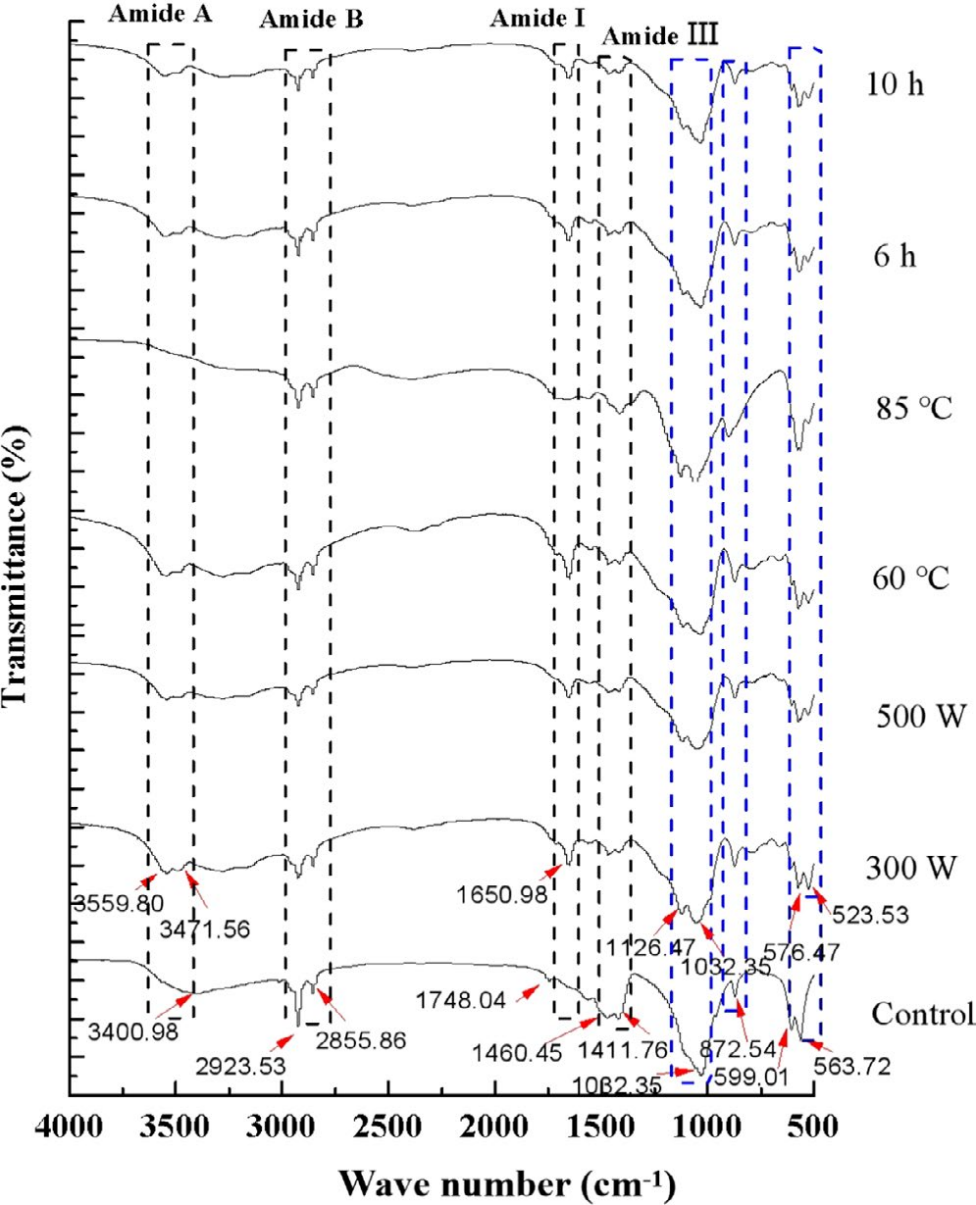
FT‐IR spectra of MFPs during ultrasonic treatment

Ultrasound waves can break down collagen by loosening or denaturing its triple helical structure (Ali et al., 2018a, 2018b). Ultrasound‐assisted extraction of hydroxyapatite from Nile tilapia fish scales disrupted the collagen structure (Sricharoen et al., 2020). Ultrasonic‐assisted extraction of nanocalcium from eggshell, which contains collagen, has also been reported (Liew et al., 2015), but the mechanism of promoting calcium release was not discussed. Therefore, this was explored in the study. The FTIR spectra of MFPs samples after ultrasonication (Figure 5) showed the characteristic bands of collagen in the untreated control samples, including peaks at 3,400.98 cm^−1^ (N‐H stretching vibration of amide A band), and 2,923.53 and 2,855.86 cm^−1^ (C‐H asymmetrical stretching and symmetrical vibration of amide B band). After ultrasonication treatment, the amide A band shifted and split into two bands at 3,471.56 and 3,559.80 cm^−1^, corresponding to the O‐H stretching mode of hydroxyapatite (Yin et al., 2016), except that the amide A band completely disappeared in the 85°C treatment samples. The intensity of the amide B bands (2,923.53 and 2,855.86 cm^‐1^) decreased to varying degrees in the U‐MFP samples. Untreated MFPs showed clear FT‐IR bands corresponding to hydroxyapatite and MCFs (Figure 5). After ultrasonic treatment, the amide A and B bands of collagen shifted and their intensity decreased, suggesting that collagen was gradually degraded, similar to the analysis of nanoscale fish bone particles after thermal treatment (Jiang et al., 2020). Peaks in the range from 1,646 to 1,652 cm^‐1^ corresponding to the C=O stretching vibration assigned to the amide I band, characteristic of collagen, were not detected in control samples, whereas the 1,652 cm^‐1^ band was observed in all U‐MFP samples, except 85°C. In addition, the amide III band (1,400–1,460 cm^‐1^) is a combination of C‐N stretching and N‐H deformation and is also characteristic of collagen. All the samples displayed the bands at 1,460.45 and 1,411.76 cm^‐1^, but their intensity decreased after ultrasonication. The amide I bands of collagen were intense and the amide III bands were attenuated in U‐MFP spectra. Both these spectral changes indicate a reduction in hydrogen bonding (Ahmed et al., 2019; Dhara, 2010). Hydrogen bonding is the main stabilizing interaction of the α‐helical peptide chains forming the triple helical structure of collagen (Bhattacharjee & Bansal, 2010), and the weakening of these interactions in U‐MFPs suggests that the collagen structure had been partially denatured.

The characteristic bands of hydroxyapatite, at 1,010–1,050 cm^‐1^ (*v*_3_ stretching mode), 960 cm^‐1^ (*v*_1_ stretching mode), 599–605 cm^‐1^ (*v*_4_ stretching mode), and 560–565 cm^‐1^ (bending mode) are assigned to PO_4_^3−^ groups (Liu et al., 2003). Control MFPs displayed bands at 1,032.35, 599.01, and 563.72 cm^‐1^. After ultrasonication, the band at 1,032.35 cm^‐1^ shifted and split into bands at 1,126.47 and 1,032.35 cm^‐1^. The bands at 599.01 and 563.72 cm^‐1^ also shifted and their separation decreased. The shifted, split, and attenuated of the PO_4_^3−^ groups in U‐MFPs spectra suggested that the hydroxyapatite crystal structure had also been partially disrupted (Yin et al., 2016). Hydroxyapatite is a three‐dimensional network of PO_4_ tetrahedra, which are linked together by columns of ninefold coordinated Ca1 atoms (Ivanova et al., 2001). The spectral changes in the PO_4_^3−^ FT‐IR bands after ultrasonic treatment indicate that PO_4_^3−^ (or calcium) escaped from hydroxyapatite and that the crystallinity of hydroxyapatite was reduced. Partly amorphous hydroxyapatite could convert into a hydrated, highly water‐soluble form, promoting the dissolution of PO_4_^3−^ groups and promoting calcium release into solution (Jiang et al., 2020; Wei et al., 2011).

#### 3.3.3 X‐ray diffraction analysis

**FIGURE 6:**
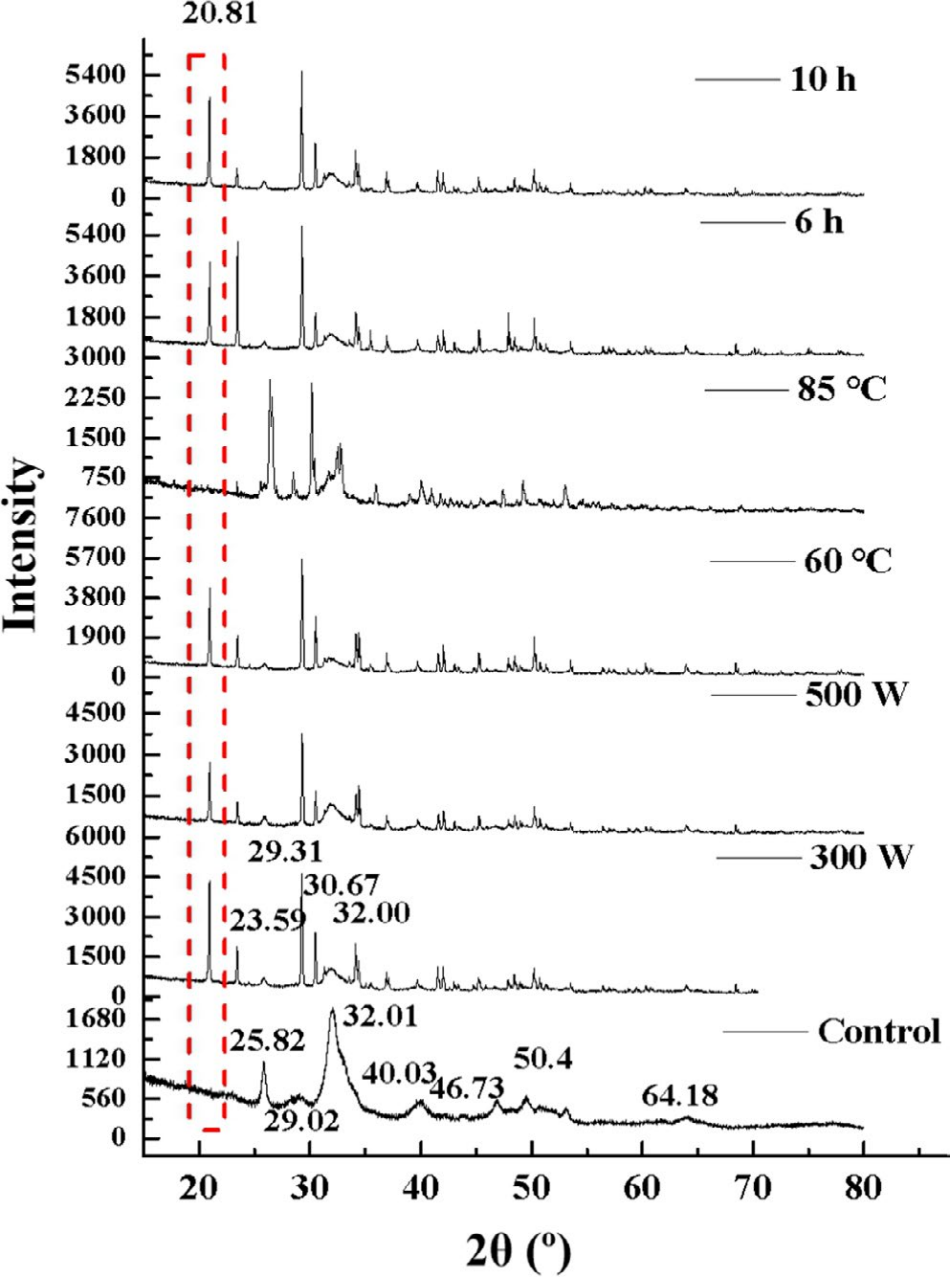
XRD spectra of MFPs during ultrasonic treatment

X‐ray diffraction was also used to detect the changes to the crystalline structure of hydroxyapatite and MCFs. XRD patterns of MFPs with or without ultrasonication are shown in Figure 6. Peaks at 2θ = 25.82°, 29.02°, 32.01°, 40.03°, 46.73°, 49.4°, and 64.18° are the characteristic peaks of hydroxyapatite (Das et al., 2018; Jiang et al., 2020). The untreated control samples exhibited the characteristic peaks of intact hydroxyapatite crystals, but no peaks of crystalline collagen, indicating that the crystallinity of collagen was disrupted by the presence of hydroxyapatite crystals. The MCFs in MFPs are tightly packed in parallel arrays and act like an adhesive, bonding hydroxyapatite, and thereby distorting the crystalline structure of the collagen, so it cannot be detected by XRD (Zhang et al., 2017). After ultrasound treatment, the characteristic peak of crystalline collagen at 21° (Nogueira et al., 2019) appeared and those of hydroxyapatite at 32.01° and 25.82° both shifted and weakened. The peak intensity and sharpness of the peak at 25.82° decreased and a new peak at 23.41° appeared. The broad peak at 32.01° greatly decreased in intensity and two sharp and intense peaks appeared at 29.28° and 30.61°. These spectral changes indicated that the crystal structure of hydroxyapatite was disrupted and some of the hydroxyapatite was released from the MFPs; thereafter, the collagen appeared to rearrange into its preferred crystalline structure. These findings are different from those for nanomilling, which also attenuated the characteristic peaks of collagen (Jiang et al., 2020).

## 4 CONCLUSION

Taken together, the findings of this study indicated that ultrasonic treatment promoted calcium release from micrometer‐scale fish bone particles (MFPs). Ultrasonication at 300 W, 60°C and for 2 h increased calcium release by 25.6% and calcium release reached 94.0% of total calcium, after 24 h ultrasonication. Overall, these findings demonstrate that ultrasonication can disrupt the crystalline structure of hydroxyapatite, promoting its dissolution, but did not denature the crystalline triple helical structure of collagen in the MCFs, only loosened and opened it up, allowing the hydroxyapatite crystals to escape. Ultrasonication may be a practical alternative to nanomilling for industrial processing of waste fish bones to produce soluble calcium as an ingredient in calcium supplements and supplemented foods.

## CONFLICT OF INTEREST

The authors declare that they do not have any conflict of interest.

## ETHICAL APPROVAL

Informed Consent: Written informed consent was obtained from all study participants.

## Data Availability

Data are available on request from the authors.
